# Constitutive overexpression of *GsIMaT2* gene from wild soybean enhances rhizobia interaction and increase nodulation in soybean (*Glycine max*)

**DOI:** 10.1186/s12870-022-03811-6

**Published:** 2022-09-09

**Authors:** Doaa Bahaa Eldin Darwish, Mohammed Ali, Aisha M. Abdelkawy, Muhammad Zayed, Marfat Alatawy, Aziza Nagah

**Affiliations:** 1 Botany Department, Faculty of Science, Mansoura University, Mansoura, 35511 Egypt; 2 Department of Biology, College of Science, Tabuk University, Tabuk, 74191 Saudi Arabia; 3 Department of Genetic Resources, Desert Research Center, Egyptian Deserts Gene Bank, North Sinai Research Station, 1 Mathaf El-Matarya St., El-Matareya, Cairo, 11753 Egypt; 4 Botany and Microbiology Department, Faculty of Science, Al-Azhar University (Girls Branch), Cairo, Egypt; 5 Botany and Microbiology Department, Faculty of Science, Menoufia University, Menoufia, Shebin El-Kom, 32511 Egypt; 6 Botany and Microbiology Department, Faculty of Science, Banha University, Qalyubia Governorate, Benha, 13518 Egypt

**Keywords:** *Glycine soja*, *Glycine max*, Root growth and nodulation, Isoflavone, Strigolactone, *GsIMaT2* gene

## Abstract

**Background:**

Since the root nodules formation is regulated by specific and complex interactions of legume and rhizobial genes, there are still too many questions to be answered about the role of the genes involved in the regulation of the nodulation signaling pathway.

**Results:**

The genetic and biological roles of the isoflavone-7-O-beta-glucoside 6″-O-malonyltransferase gene *GsIMaT2* from wild soybean (*Glycine soja*) in the regulation of nodule and root growth in soybean (*Glycine max*) were examined in this work. The effect of overexpressing *GsIMaT2* from *G. soja* on the soybean nodulation signaling system and strigolactone production was investigated. We discovered that the *GsIMaT2* increased nodule numbers, fresh nodule weight, root weight, and root length by boosting strigolactone formation. Furthermore, we examined the isoflavone concentration of transgenic *G. max* hairy roots 10 and 20 days after rhizobial inoculation. Malonyldaidzin, malonylgenistin, daidzein, and glycitein levels were considerably higher in *GsMaT2*-OE hairy roots after 10- and 20-days of *Bradyrhizobium japonicum* infection compared to the control. These findings suggest that isoflavones and their biosynthetic genes play unique functions in the nodulation signaling system in *G. max*.

**Conclusions:**

Finally, our results indicate the potential effects of the *GsIMaT2* gene on soybean root growth and nodulation. This study provides novel insights for understanding the epistatic relationship between isoflavones, root development, and nodulation in soybean.

**Highlights:**

* Cloning and Characterization of 7-O-beta-glucoside 6″-O-malonyltransferase (*GsIMaT2*) gene from wild soybean (*G. soja*).

* The role of *GsIMaT2* gene in the regulation of root nodule development.

*Overexpression of *GsMaT2* gene increases the accumulation of isoflavonoid in transgenic soybean hairy roots.

* This gene could be used for metabolic engineering of useful isoflavonoid production.

**Supplementary Information:**

The online version contains supplementary material available at 10.1186/s12870-022-03811-6.

## Background

The symbiotic interaction between soybean roots and *B. japonicum* bacteria leads to the formation of unique structures Known as root nodules. Hosted inside the root nodule, rhizobia can transform the molecular nitrogen gas (N_2_) from the atmosphere into ammonia (NH_3_), which will be readily available to the plant, and for this exchange of benefits deal, rhizobia is amended with plant carbohydrates [1, 2]. Various factors regulate root nodule formation, such as certain plant hormones, some metabolic enzymes, and definite transcription factors from the approach of the nodulation signal to nodule initiation, development, and maturation [3, 4]. Furthermore, several genes related to secondary metabolism (e.g., Phenylpropanoids and isoflavonoids biosyntheses) were identified by microarray analysis from *Lotus japonicu* nodule with higher frequency in nodule parenchyma (NP) and nodule vascular bundle (NC) compared with un-nodulated root [5]. Previously, we found that the overexpression of *GmIMaTs* from soybean and *MtMaTs* genes from *Medicago sativa* led to a dramatic increase in isoflavonoid malonates, which consider signals to straightforward the symbiotic interaction of legume plants and rhizobia [6, 7].

Isoflavone components are considered the largest ecophysiologically active secondary metabolites, with various structures almost exclusively represented in legumes [8]. Legumes are one of the most vital food staff worldwide; leguminous species (e.g., soybean, snow pea, lentil, lupine mung bean, hairy vetch, alfalfa, medicago, white clover, and red clover) produce various isoflavones compounds, which play role(s) as auxin transport regulators, plant defense, plant growth, acting as signals to regulate the symbiotic interaction of legume plants and rhizobia [6, 9–15]. The genome of some Legumes plants, such as soybean and Medicago has several MaT homologous; some genes (e.g., *MtMaT1, MtMaT4, MtMaT5,* and *MtMaT6*) were characterized from *M. truncatula* and other genes (e.g., *GmIMaT1, GmIMaT3, GmMT7,* and *GmIF7MaT*) were characterized from *G. max* [6, 16–19]. These previous genes use malonyl- CoA as the only acyl donor to convert glycitin, genistin, and daidzin compounds to glycitin 6-o-malonates, genistin, and daidzin [6, 16–19]. In an endeavor to understand the ability of soybean plants to generate various types and amounts of isoflavone, we found many characterized flavonoid malonyltransferase genes in the soybean genome that different studies tried to characterize some of them [6, 16–19]. We here report a new malonyltransferase gene *GsIMaT2* from wild soybean (*G. soja*). Various studies have shown that the isoflavonoid malonates stimulate the expression of Nod factor-encoding genes that are involved in nodulation signaling, such as *GmNRF1α, GmNRF5α, GmNSP1α, GmNSP2α, GmDMI2α* and *GmDMI3β* in domesticated soybean [16–19]. Moreover, strigolactones (SLs) are a class of hormones widely present in most plant species such as Arabidopsis, Pea, Rice, Petunia, and soybean [20–24]. Strigolactones (SLs) have different physiological roles correlated to root growth and development, branching of the shoot, and mycorrhiza and root nodules in legumes [24–26]. Earlier studies have illustrated that strigolactone genes detected in the root of soybean and alfalfa seedlings enhance nodulation by inducing the expression of Nod genes in rhizobial bacteria [4, 24, 27, 28].

Over the years, the transformation of cultivated soybean (*G. max*) hairy roots using *Agrobacterium rhizogenes* has become a powerful way to characterize proteins-encoding genes involved in root biological roles such as plant-microbe communication, nutrient uptake, and hormone transport [29]. We have successfully used this system to clarify the role of two More Axillary Growth genes (*GmMAX1a* and *GmMAX4a*) in soybean nodulation [28]. This work offers a functional characterization of the wild-type *G. soja* isoflavone malonyl transferase 2-encoding gene *GsIMaT2*. The results disclosed its association with flavonoid and isoflavonoid biosynthesis, and rhizobial nodulation in domesticated *G. max*. The inclusion methodologies that were employed to reach this goal are the following: (i) overexpression of (*GsIMaT2*) gene in the domesticated soybean hairy roots; (ii) inspecting nodulation and root growth characters after inoculation with *B. japonicum*. (iii) Profiling isoflavonoids in transgenic *G. max* hairy roots by HPLC (iv) Monitoring the transcription of genes implicated in nodulation signaling and strigolactones biosynthesis by qRT-PCR. Interestingly, our findings support the significance of the *GsIMaT2* in rhizobial infection by elucidating the links between nodulation-signaling genes, strigolactone-biosynthesizing genes, and *GsIMaT2*.

## Results

### Identification of *GsIMaT2* gene from *G. soja* plant genomics

The *GsIMaT2* was retrieved from the wild soybean genome by managing a BLASTP search against the *G. soja* genome using other isoflavone-7-O-beta-glucoside 6″-O-malonyltransferase proteins from *G. max, L. albus,* and *M. truncatula* as queries to authenticate authentic homology. This attitude recognized various proteins firmly correlated to *GsIMaT2*. The obtained sequences were put forward for phylogenetic analysis (Additional file 1**: **Fig. S1). Our retrieved *GsIMaT2* product is clustered within a monophyletic group with only other isoflavone malonyltransferases from either *G. max* or *G. soja*. Besides, the phylogenetic analysis ratifies the close evolutionary relationship between *GsIMaT2* and *GmIMaT2* (Additional file 1: Fig. S1). From multiple sequence alignment analyses by CLUSTALW (https://www.genome.jp/tools-bin/clustalw), and from the prediction of protein secondary structure, we found that the *GsIMaT2* has two amino acid differences from *GmIMaT2* at Q75L and D192Y (Additional files 2 and 3**: **Fig. S2 and S3). The putative expression analyses of the *GsIMaT2* were done as of its orthologous *Glyma.18G258000* from *G. max* across twenty-eight soybean tissues after inoculation and fertilization (Root Hair 12HAI, Roothair_12HAImock, Root Hair 24 HAI, Roothair_24HAImock, Root Hair 48 HAI, Roothair_48HAImock, Root Hair 48 HAI Stripped, SAM, Flower, Green_Pods, Leaves, Nodule, Root, Root_tip, Young Leaf, Flower, One CM Pod, Pod Shell (10-13 DAF), Pod Shell (14 - 17 DAF), Nodule, Root, Seed 10 - 13 DAF, Seed 14 - 17 DAF, Seed 21 DAF, Seed 25 DAF, Seed 28 DAF, Seed 35 DAF, and Seed 42 DAF) using the e-Plant Soybean database (http://bar.utoronto.ca/eplant_soybean/). Remarkably, the highest expression levels of *Glyma.18G258000* were found in the Root tip, Root Hair 48 HAI, Root hair_48HAImock, and Root Hair 24 HAI. Also, the highest expression level of our target gene was observed at Root, Seed 35 DAF, Seed 42 DAF, and Seed 28 DAF (Additional file 4**: **Fig. S4A and B). These results agree with [6, 16, 24, 30–34], which reported that higher expression levels of isoflavonoid genes such as, *GmMT7*, *GmIMaT1,* and *GmIMaT3* were detected in roots and seeds. In plants, the isoflavone-7-O-beta-glucoside 6″-O-malonyltransferase (*GsIMaT2*; EC: 2.3.1.115) gene plays essential roles in two important pathways isoflavonoid biosynthesis (KGEE:map 00943; https://www.kegg.jp/pathway/map=map00943&keyword=2.3.1.115) and Flavone and flavonol biosynthesis (KEGG:map 00944; https://www.kegg.jp/pathway/map=map00944&keyword=2.3.1.115) which are responsible for generating several structures from isoflavonoids, Flavone and flavonol [31, 35]. In these two pathways, the isoflavone malonyl transferase enzyme can use both malonyl-CoA (CPD:C00083) and biochanin A 7-O-beta-D-glucoside (CPD:C05376) as substrates for generating various isoflavonoid components such as Medicarpin 3-O-glucoside-6′-malonate (CPD:C16224); (−)-Maackiain-3-O-glucosyl-6″-O-malonate (CPD:C16231); Formononetin7-O-glucoside-6″-O-malonate (CPD:C16222); Malonyldaidzin (CPD:C16191); Malonylglycitin (CPD:C16197); Biochanin A 7-O-beta-D-glucoside 6″-O-malonate (CPD:C12625); Malonylgenistin (CPD:C16192) and Malonylapiin (CPD:C05622) [31, 35]. These previous isoflavones compounds also play a key role in plant–bacteria interactions by intermediating the symbiosis between legumes plants and N_2_-fixing bacteria [6, 8]. In relevance to plant-microbe interaction, we can classify the Rhizobium genes into two groups, the first group which related to the synthesis of bacterial cell surfaces such as β-1,2-glucans (*ndv* genes), lipopolysaccharides (*lps* genes), capsular polysaccharides of K antigens, and exopolysaccharides (*exo* genes) [6, 8, 9]. While the second group comprises nodulation (nod) genes. Isoflavonoids from the legumes act as a key factor in inducing the activation of rhizobial nodulation genes through two steps; in the first step the flavonoids released from plant roots form a complex with the NodD protein to induce the transcription of bacterial nod genes.

On the other hand, in the second step, a Rhizobium soil bacterium produces Nod factors (lipooligosaccharide signals) that promote the root responses through various structural nod genes [6, 8, 9, 13, 16, 24]. Consequently, the subcellular localization of the *GsIMaT2* products was predicted using the Cell eFP browsers (http://bar.utoronto.ca/cell_efp/cgi-bin/cell_efp.cgi) from its closest orthologous protein in Arabidopsis. From this analysis, the *GsIMaT2* localizes chiefly in the cytosol, followed by the endoplasmic reticulum, mitochondria, nucleus, plastids, and Golgi (Additional file 4**: **Fig. S4C).

### Effect of *GsIMaT2* gene over-expression on soybean nodulation after *B. japonicum* (USDA110) infection

**Fig. 1:**
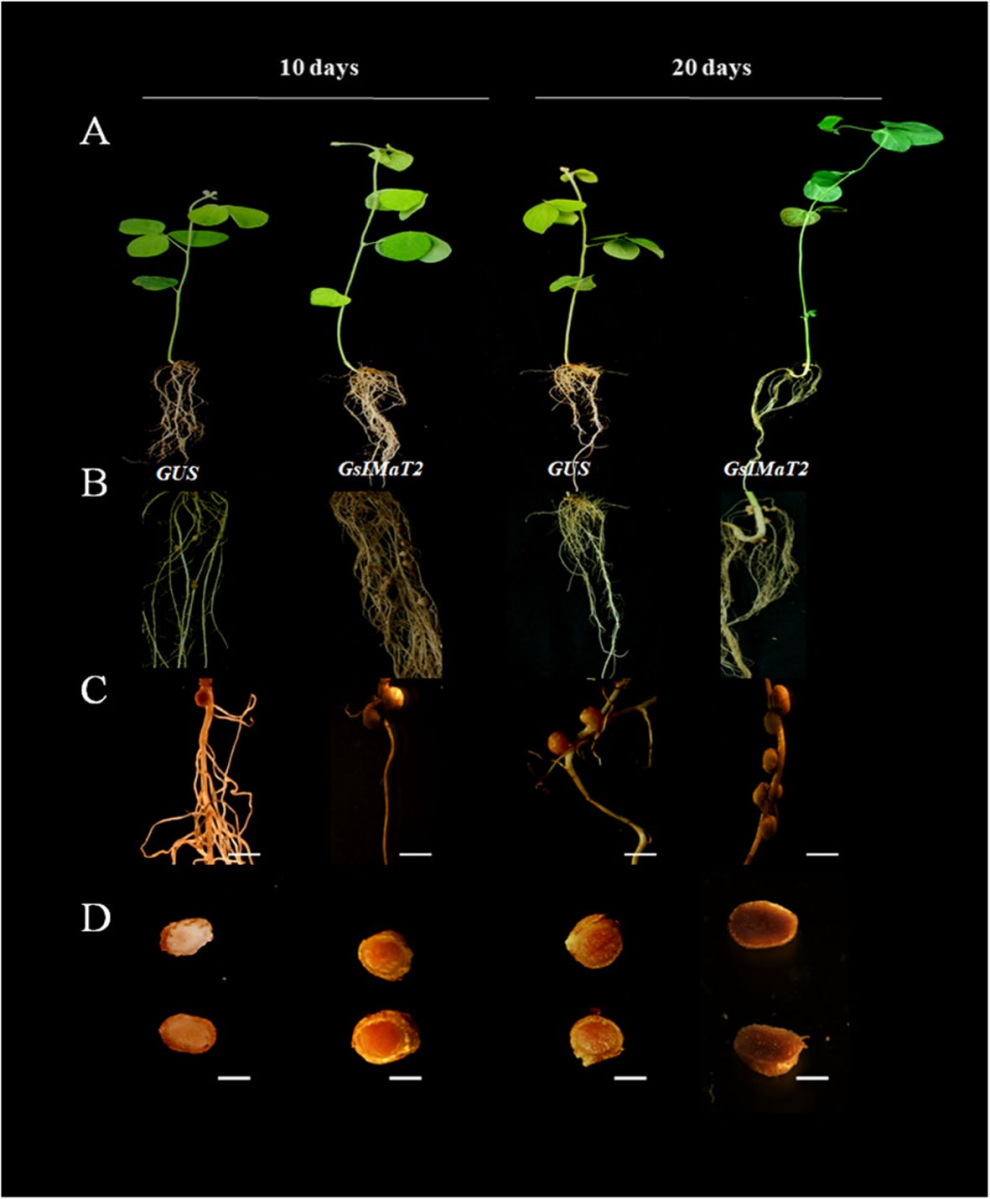
Effect of *GsIMaT2* gene overexpression on soybean root nodulation. Roots and nodules were examined on the 10th and 20th days after rhizobia were inoculated with *B. japonicum* strain USDA110. Composite plants were generated by transformation with the K599 vector harboring overexpression cassettes for GUS (control) and *GsIMaT2*. Roots were inoculated with rhizobia. **A** Root and shoot phenotypes of 10 and 20-d-old G. max plants. **B** Locations where nodules formed on hairy roots overexpressing 10 and 20-days after rhizobial inoculation. **C** Nodules developed on secondary roots. **D** Cross-sections of *G. max* nodules. Photographs in **C** and **D** were taken with a DP-73 microscope camera set (Olympus, Tokyo, Japan). Scale bars in C and D = 500 μm

**Fig. 2:**
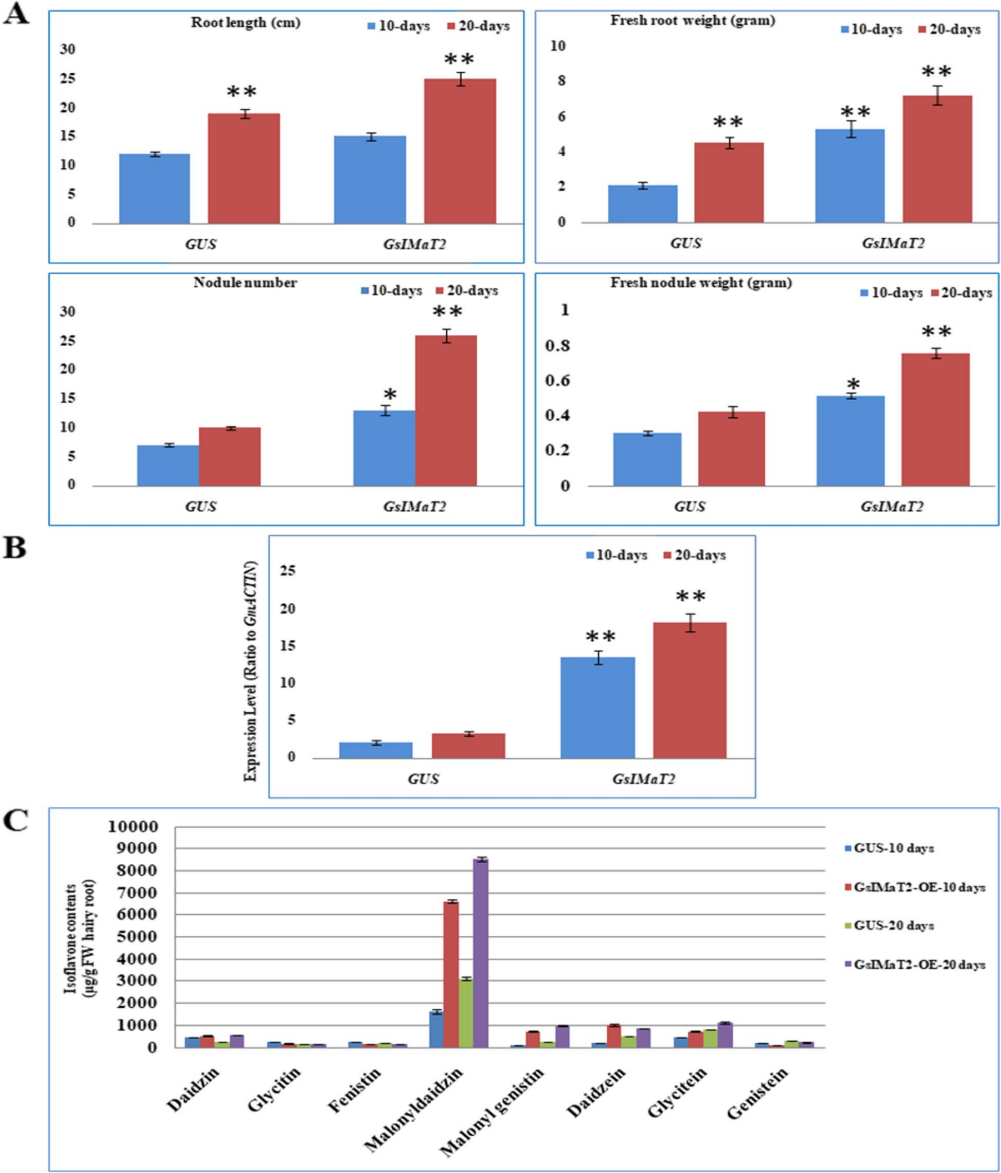
Effects of *GsIMaT2* gene overexpression on root growth and nodule development at 10 and 20 days of rhizobia inoculation. **A** In vivo root length (cm), fresh root weight (gram), nodule numbers, and fresh nodule weight (gram) were examined (*n* = 10-12). The blue and red columns represent the effect of gene overexpression 10- and 20-days after rhizobial inoculation. Data are presented as means ± SD, and statistical significance is based on Student’s t-test (*P < 0.05; ** *P* < 0.01) with GUS-overexpressing hairy roots as the control. **B** Quantitative RT-PCR for in vivo hairy roots after 10- and 20-days from *B. japonicum* (USDA110) infection. The error bars indicate the SD of three qRT-PCR biological replicates. **C** HPLC analysis for profiling the isoflavonoids in the transgenic *G. max* hairy roots after 10 and 20 days post-inoculation

To evaluate the effect of *GsIMaT2* gene in transgenic *G.max* hairy roots, the *GsMaT2* gene from *G. soja* was cloned and over-expressed on soybean nodulation upon 10- and 20-days after *B. japonicum* (USDA110) infection. Chimeric *G. max* plants were grown in vermiculite soil then the hairy roots were inoculated with *B. japonicum* to examine the impacts of *GsIMaT2* on soybean nodulation and root phenotypes after 10- and 20-days after rhizobial infection (DAI) as shown in Fig. 1A-D. The following root and nodule characteristics were investigated: root length (cm), fresh root weight (gram), nodule number, and fresh nodule weight (gram), as indicated in Fig. 2A. 10- and 20-days after inoculation, qRT-PCR was used to confirm the expression level of *GsIMaT2*, which showed considerable overexpression as compared to the GUS control. (Fig. 2B). Our results reveal that *GsIMaT2* overexpression improved root length and fresh root weight **(**Fig. 2A). Furthermore, as compared to GUS lines, overexpression of the *GsIMaT2* gene also resulted in higher nodule numbers and significantly raised nodule fresh weight for a given amount of root (Fig. 2A). As a brief outcome, overexpression of the *GsIMaT2* gene may alter soybean nodulation 10- and 20-days after rhizobia infection. (Fig. 1A-D and Fig. 2A). Our findings suggest that the *GsIMaT2* gene may play a sustained role in the *B. japonicum*-induced soybean nodulation signaling pathway.

### *GsMaT2* overexpression changed isoflavone profiles in transgenic soybean hairy roots

To explore the consequence of the wild-type isoflavone malonyltransferase *GsIMaT2* on isoflavone malonylation, quantitative HPLC was performed to analyze various isoflavones in transgenic *G. max* hairy roots (Fig. 2C). The analysis revealed that malonyldaidzin, malonylgenistin, daidzein and glycitein levels were significantly increased in *GsMaT2-OE* hairy roots after 10- and 20-days of *B. japonicum* infection compared with the control (Fig. 2C). While, glycitin, fenistin and genistein were significantly decreased in *GsMaT2-OE* hairy roots after 10- and 20-days after *B. japonicum* infection as compared with the control (Fig. 2C). On the other hand, daidzin was increased with a few levels after 10-days from infection, and exhibited an ~ 1.5-fold increase after 20-days of infection in comparison with the control (Fig. 2C). These findings results are in accordance with [6, 16] they revealing that the overexpression of *GmIMaT1, GmIMaT2*, and *GmIMaT3* increased the concentrations of malonyldaidzin, malonylgenistin, daidzein and glycitein in transgenic *G.max* hairy roots.

### Overexpression of *GsIMaT2* gene in transgenic soybean hairy roots altered the expression of nodulation and SL biosynthesis genes

The creation of root nodules takes place when legume roots and rhizobia recognise one other, resulting in the formation of symbiotic interactions known as infection foci [36].

**Fig. 3:**
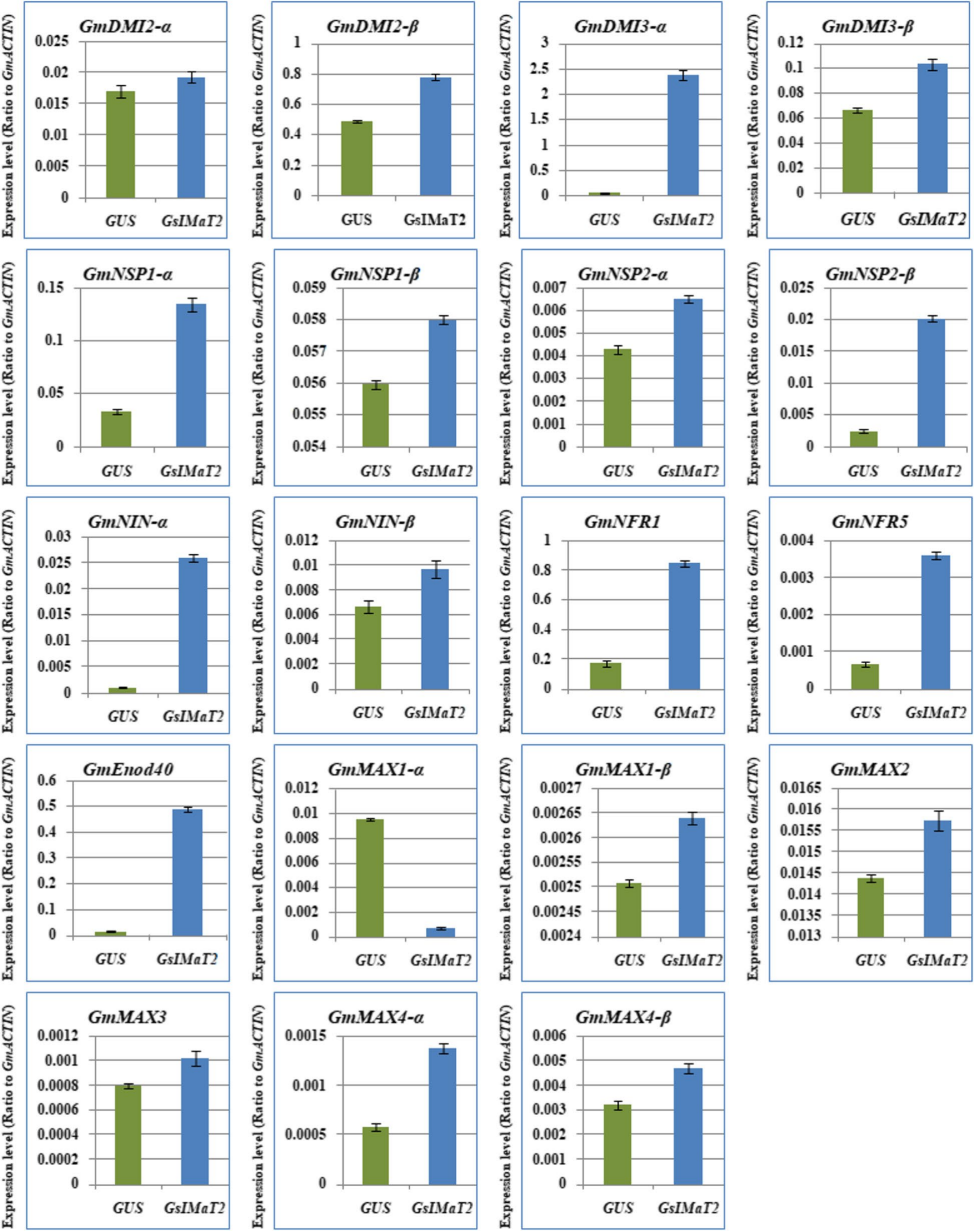
Expression profiles of nodulation and SL biosynthesis genes under the effect of *GsIMaT2* gene overexpression in soybean transgenic hairy roots after 10 days of rhizobia inoculation. Gene expression was analyzed using quantitative real-time PCR compared to GUS as a control. The housekeeping *GmB-ACTIN* gene was used as an internal reference gene for expression normalization. The error bars indicate the SD of three qRT-PCR biological replicates

In order to decide whether the *GsIMaT2* gene influences rhizobial infection and nodule formation, we analyzed the transcript levels of the induction signaling genes and SLs biosynthesis in soybean hairy roots after 10 day of *B. Japonicum* infection to have a better understanding of the role of *GsIMaT2* during symbiotic nitrogen fixation in legumes. For that purpose, we analyzed the expression levels of 19 selected genes, including early nodulation signaling genes such as *GmDMI2a, GmDMI2B, GmDMI3a, GmDMI3b, GmNSP1a, GmNSP1B, GmNSP2a, GmNSP2B, GmNINa, GmNINB, GmNRF1, GmNRF5,* and *GmEnod40*, and SL synthetic genes such as *GmMAX1a, GmMAX1B, GmMAX2, GmMAX3, GmMAX4a,* and *GmMAX4B*. Our outcomes manifested that the chosen nineteen SLs biosynthesis and signaling pathway genes were divergently expressed in the hairy roots 10DAF (Fig. 3). Expression levels of *GmDMI2a, GmDMI2B, GmDMI3a, GmDMI3b, GmNSP1a, GmNSP1B, GmNSP2a, GmNSP2B, GmNINa, GmNINB, GmNRF1, GmNRF5, GmEnod40, GmMAX1B, GmMAX2, GmMAX3, GmMAX4a,* and *GmMAX4B* genes were highest in hairy roots overexpressing *GsIMaT2*. While the *GmMAX1a* gene was at the lowest expression level in hairy roots overexpressing *GsIMaT2* (Fig. 3). The expression patterns of nodulation signaling and SLs biosynthesis genes in hairy roots overexpressing *GsIMaT2* gene during the first 10 days of root nodulation suggested that, overall, *GsIMaT2* play important roles during nodulation signaling and the early stages of nodule development.

### Overexpressing *GsIMaT2* gene changed the expression of nodulation and SL biosynthesis genes in soybean nodules upon *B. japonicum* (USDA110) infection

**Fig. 4:**
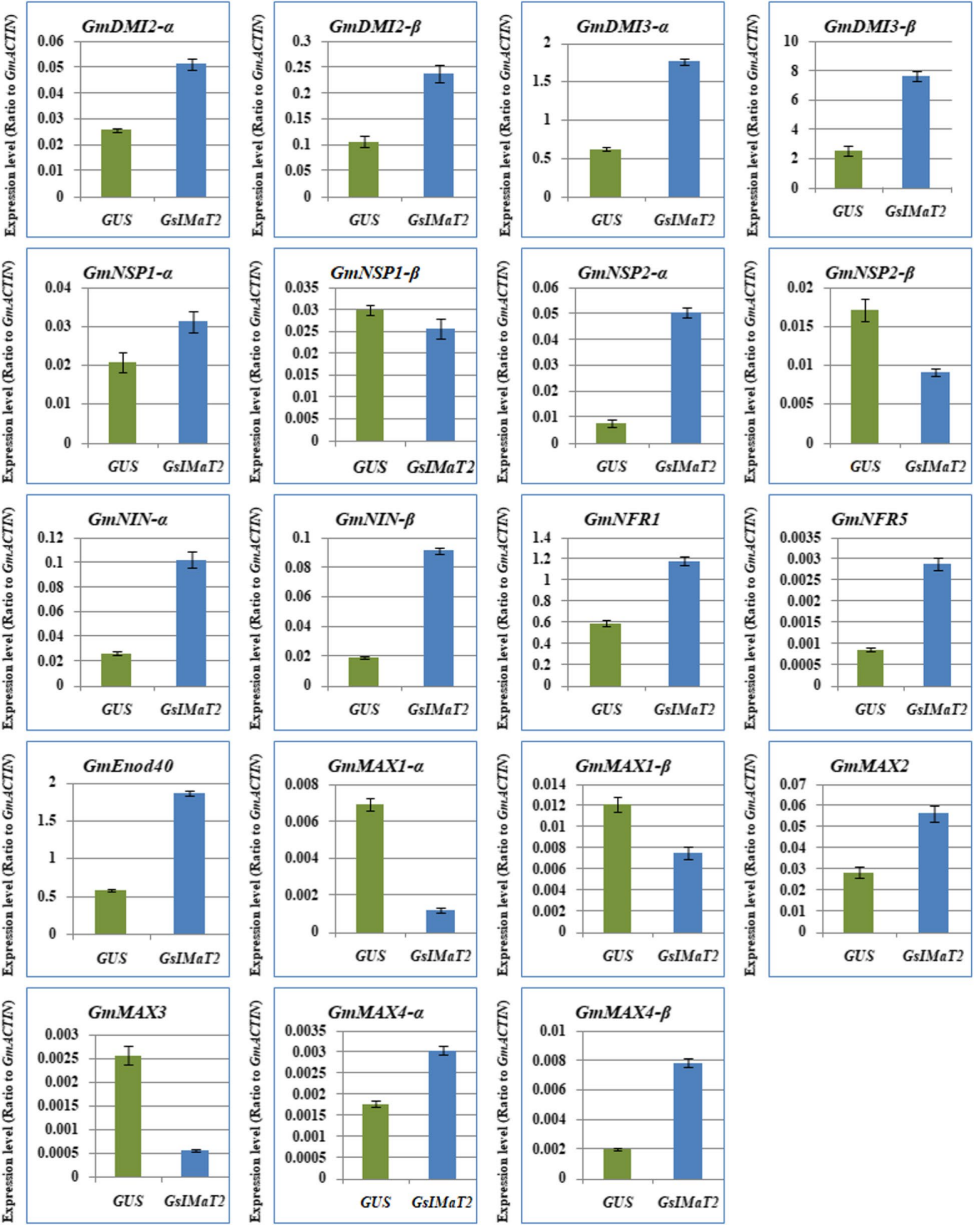
Expression profiles of nodulation and SL biosynthesis genes under the effect of *GsIMaT2* gene overexpression in soybean nodules after 10 days of rhizobia inoculation. Gene expression was analyzed using quantitative real-time PCR compared to GUS as a control. The housekeeping *GmB-ACTIN* gene was used as an internal reference gene for expression normalization. The error bars indicate the SD of three qRT-PCR biological replicates

**Fig. 5:**
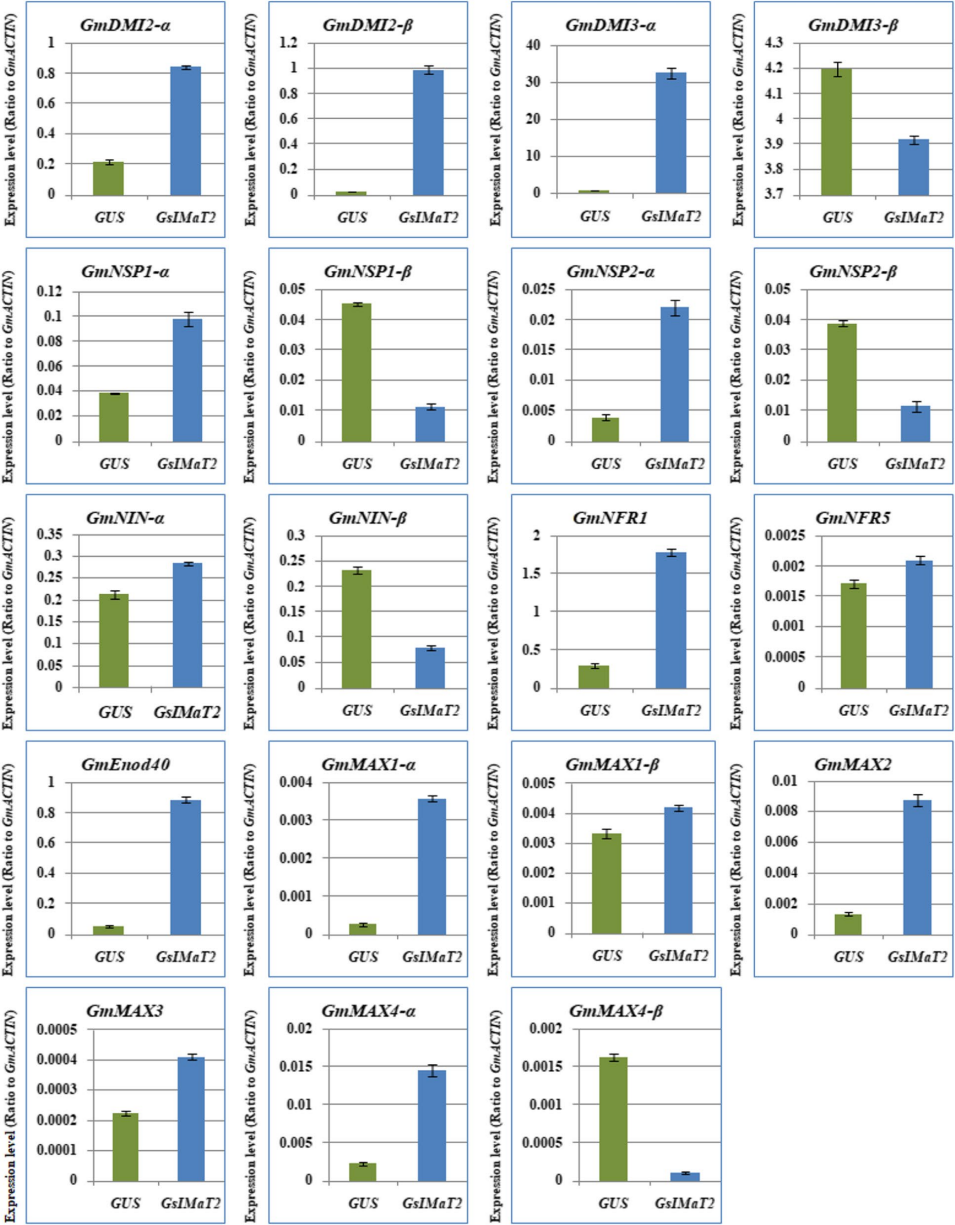
Expression profiles of nodulation and SL biosynthesis genes under the effect of *GsIMaT2* gene overexpression in soybean transgenic hairy roots after 20 days of rhizobia inoculation. Gene expression was analyzed using quantitative real-time PCR compared to GUS as a control. The housekeeping *GmB-ACTIN* gene was used as an internal reference gene for expression normalization. The error bars indicate the SD of three qRT-PCR biological replicates

**Fig. 6:**
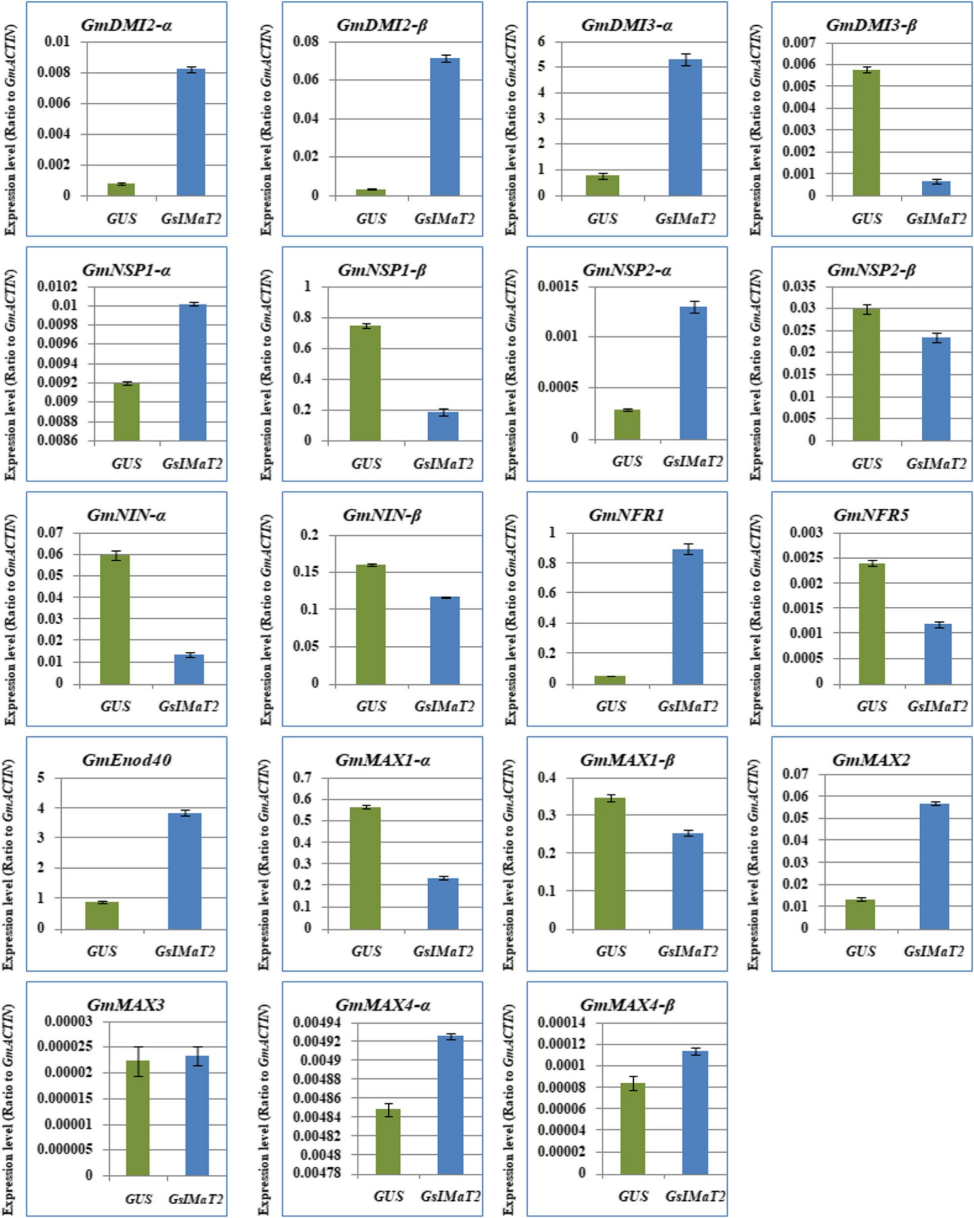
Expression profiles of nodulation and SL biosynthesis genes under the effect of *GsIMaT2* gene overexpression in soybean nodules after 20 days of rhizobia inoculation. Gene expression was analyzed using quantitative real-time PCR compared to GUS as a control. The housekeeping *GmB-ACTIN* gene was used as an internal reference gene for expression normalization. The error bars indicate the SD of three qRT-PCR biological replicates

To determine if *GsIMaT2* overexpression plays a functional role during rhizobial infection at the early stages of nodule formation and development, we studied the effect of *GsIMaT2* overexpression on the expression of signaling genes and SLs biosynthesis in nodulation 10 DAI by *B. japonicum*. As a result, the expression levels of the same nineteen previously identified genes, early nodulation signaling, and SL biosynthesis genes were evaluated and analyzed. These findings illustrated that at 10 DAI, the previously mentioned genes were differently activated in nodules (Fig. 4). Intriguingly, the expression levels of *GmDMI2a, GmDMI2B, GmDMI3a, GmDMI3b, GmNSP1a, GmNSP2a, GmNINa, GmNINB, GmNRF1, GmNRF5, GmEnod40, GmMAX2, GmMAX4a,* and *GmMAX4B* genes were highest in nodules overexpressing *GsIMaT2*. Conversely, *GmNSP1B, GmNSP2B, GmMAX1a, GmMAX1B,* and *GmMAX3* transcription levels were markedly declined in nodules by overexpressing *GsIMaT2* (Fig. 4). This result indicates that *GsIMaT2* has a censorious symbiotic role during nodulation signaling and the early stages of nodule formation. To investigate the sustained effects of *GsIMaT2* gene overexpression on root and nodule development, we looked at the expression of nodulation signaling and SLs biosynthesis genes in hairy roots and nodules of soybean at 20 DAI. Moreover, qRT-PCR was used to examine the expression levels of the same collection of nodulation signaling and SLs biosynthesis genes. A 20 DAI, the prior genes were differentially induced in hairy roots and nodules, according to the findings (Figs. 5 and 6). As shown in the result (Fig. 5), the expression of *GmDMI2a, GmDMI2B, GmDMI3a, GmNSP1a, GmNSP2a, GmNINa, GmNRF1, GmNRF5, GmEnod40, GmMAX1a, GmMAX1b, GmMAX2, GmMAX3,* and *GmMAX4a* genes were enormously increased in hairy roots overexpressing *GsIMaT2* compared with GUS as control at 20 DAI. However, the expression levels of *GmDMI3B, GmNSP1B, GmNSP2B, GmNINB,* and *GmMAX4B* genes were decreased in hairy roots overexpressing *GsIMaT2* (Fig. 5), signifying the role of *GsIMaT2* at a late stage of rhizobial infection. In addition, the expressions of *GmDMI2a, GmDMI2B, GmDMI3a, GmNSP1a, GmNSP2a, GmNRF1, GmEnod40, GmMAX2, GmMAX3, GmMAX4a,* and *GmMAX4B* genes were the highest in nodules overexpressing *GsIMaT2* compared with GUS as control at 20 DAI.

While, the expression levels of *GmDMI3B, GmNSP1B, GmNSP2B, GmNINa, GmNINB, GmNRF5, GmMAX1a,* and *GmMAX1B* genes were the lowest in nodules overexpressing *GsIMaT2* (Fig. 6), suggesting diverse roles of this *GsIMaT2* gene during hairy soybean roots and nodules development. Consequently, these data indicate that *GsIMaT2* expression orchestrates nodulation signaling and SL biosynthesis genes in hairy roots and nodules at 20 DAI. Furthermore, these findings show that *GsIMaT2* has a long-term effect on root development, nodule formation, and nodule development, even in mature root systems undergoing active symbiotic nitrogen fixation.

## Discussion

### Characterization, putative expression patterns, and putative subcellular localization of *GsIMaT2* from *G. soja* plant

Wild and cultivated soybean (*G. soja* [Sieb. and Zucc.] and *G. max* [L.] Merr) are considered one of the oldest polyploid (pa leopolyploid) plants and one of the most vital food crops worldwide. Soybean isoflavonoids were analyzed several decades ago and were found to contain several of aglycon and glucoside isoflavonoids components, such as daidzein, genistein, glycitein, aglycones, malonyldaidzin, malonylgenistin, and malonylglycitin [6, 37, 38]. Despite this, only a limited number of recent reports describe the role and function of isoflavonoid genes in soybean root growth and nodulation [8, 19, 30, 39]. In this study, we identified the isoflavone-7-O-beta-glucoside 6″-O-malonyltransferase (*GsIMaT2*) gene in the wild soybean genome by BLAST search using the putative isoflavone-7-O-beta-glucoside 6″-O-malonyltransferase gene from *G. max, L. albus,* and *M. truncatula*. Phylogenetic analysis showed that the close homology to *GsIMaT2* from *G. soja* is *GmIMaT2* from *G. max* (Additional file 1: Fig. S1). To recognize *GsIMaT2* physiological roles, its expression patterns in twenty-eight different tissues following inoculation and fertilization based on their increased resemblance to *Glyma.18G258000* gene from *G. max* were identified. This *GsIMaT2* gene was detected in all the tissues and predominantly expressed after inoculation in (Root_tip, Root Hair 48 HAI, Root hair_48HAImock, and Root Hair 24 HAI), and after fertilization in (Root, Seed 35 DAF, Seed 42 DAF, and Seed 28 DAF), which are nearly similar to these homologous genes *GmMT7, GmMaT1, GmMaT2, GmMaT3* and *GmMaT4* from soybean (Additional file 4**: **Fig. S4 A and B) [6, 16, 30–34]. Moreover, putative subcellular localization studies based on Arabidopsis protein localization for recognized synthesis sites from the Cell eFP database (http://bar.utoronto.ca/cell_efp/cgi-bin/cell_efp.cgi) revealed that the *GsIMaT2* presents mainly in the cytosol and endoplasmic reticulum (Additional file 4**: **Fig. S4C). These in silico results align with earlier studies that exhibited cytosol and endoplasmic reticulum as the main loci for isoflavonoids [6, 7, 33, 40–42]. The putative expression patterns and putative subcellular localization of *GsIMaT2* underscore the possible roles of (iso) flavonoids in yielding flavonoids found at infection sites and during infection to attract rhizobia and establish nodulation [39]. Therefore, cloning the full-length cDNA of *GsIMaT2* and examining its role in soybean root and nodule development through overexpressing in hairy root systems is crucial to proving this hypothesis (Fig. 1A-D). The results demonstrated that this gene plays a significant role in increasing root length, fresh root weight, nodule number, and fresh nodule weight, compared to the GUS control in transgenic *G. max* hairy roots (Fig. 2A). Isoflavonoids and their derivatives genes are reported to affect legume nodulation, and well-documented to function in *M. truncatula* and *G. max* root nodule.

### Overexpression of *GsMaT2* gene changed accumulation of isoflavonoid in transgenic soybean hairy roots

Exhilaratingly, we revealed that the overexpression of *GsMaT2* enhanced isoflavonoid amassing in transgenic soybean hairy roots (Fig. 2C). From our results, we found that the malonyldaidzin, malonylgenistin, daidzein and glycitein were significantly increased in *GsMaT2-OE* hairy roots after 10- and 20-days from *B. japonicum* infection compared with the GUS control (Fig. 2C). Particularly, isoflavonoid compounds such as glyceollins, daidzein, malonyldaidzin, genistein, and malonylgenistin were reported to affect plant growth, nodule formation and interaction with other microbial communities [6, 7, 12, 14, 15]. However, plenty of evidence showed the role of isoflavonoid like daidzein and genistein that are secreted from roots in root–bacteria symbiotic interaction, which starts from secreting and transporting to the plasma membrane of root cells [43]. Then inducing the expression of Nod genes in rhizobial bacteria to form infection threads and nodules formation in root cortical [2, 17]. Likewise, isoflavonoid compounds have been shown to affect nodule formation and root hair patterning in soybean and Medicago. For example, isoflavonoid genes such as *MtMaT1, MtMaT4, MtMaT5, MtMaT6, GmIMaT1, GmMaT2,* and *GmIMaT3* can affect nodule and root development in soybean and medicago transgenic roots, likely through modulating the accumulation of isoflavonoid [6, 16, 41, 42]. Therefore, it is not surprising that the overexpression of the *GsMaT2* gene in transgenic soybean hairy roots showed similar effects on the isoflavonoid accumulation and nodules formation.

### Effect of *GsMaT2* overexpressing in hairy roots growth and soybean nodulation

**Fig. 7:**
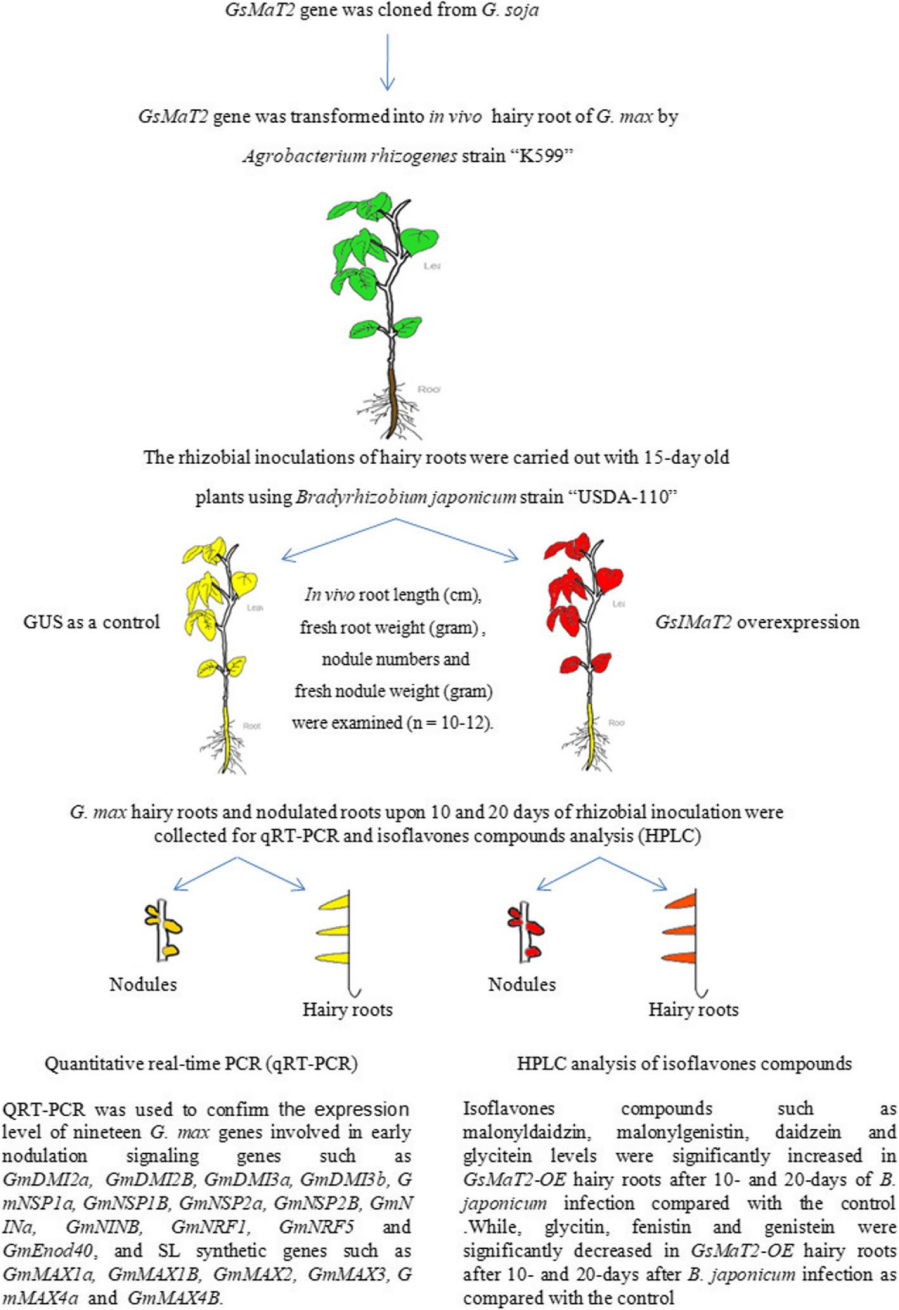
Summary of key steps used to get the overall findings. These steps were used to study the roles of *GsMaT2* from wild soybean (*G. soja*) in root growth, root nodulation, isoflavones accumulation, expression levels of nodulation signaling, and SL biosynthesis genes in cultivated soybean (*G. max*). Plant cartoon form and plant parts were obtained from the soybean eFP Browser site (http://bar.utoronto.ca/efpsoybean/cgi-bin/efpWeb.cgi)

To shed light on the role of *GsMaT2* overexpressing in controlling hairy roots growth and nodulation, we investigated the effect of *GsMaT2* gene overexpression on the expression levels of nodule signaling and SLs biosynthesis genes in hairy roots and nodules of transgenic soybean upon 10 and 20-days after *B. japonicum* inoculation. Quantitative real-time (qRT) PCR was used to determine the expression levels of nineteen selected genes (*GmDMI2a, GmDMI2B, GmDMI3a, GmDMI3b, GmNSP1a, GmNSP1B, GmNSP2a, GmNSP2B, GmNINa, GmNINB, GmNRF1, GmNRF5, GmEnod40,GmMAX1a, GmMAX1B, GmMAX2, GmMAX3, GmMAX4a* and *GmMAX4B*) that are related with nodulation signaling and SL synthetic genes (Fig. 3, 4, 5 and 6). Our findings revealed that several of these genes were significantly activated by the overexpression of *GsMaT2* gene in both of hairy roots and root nodules. In context, each one of these previous genes has a role(s) in rhizosphere plant-microbe interactions and nodule development. For example, Nod factor receptor genes (NFR1 and NFR5) central nodulation signaling gene in legume that specifically recognizes and binds to compatible, species-specific Nod factors produced by rhizobia [44–48]. These results are supported by [3, 16, 24, 49–52] that findings highlight the importance of Nod factor receptors (NFRs) genes from *M. truncatula, L. japonicus,* and *G. max* for interact with root-rhizobia, activate early nodulin gene expression and nodule organogenesis. Furthermore, Nodule inception genes (*GmNINa* and *GmNINb*) are early key regulators of nodule organogenesis and infection thread formation [53–55]. Moreover, the transcriptional regulators’ Nodulation Signaling Pathway1 (*NSP1*) and *NSP2* are essential for inducing and activating the expression of Nodule Inception (*NIN*), Early Nodulin coding genes (*ENOD11* and *ENOD40*), and Ethylene Response Factor Required for Nodulation1 coding gene (*ERN1*) throughout Rhizobial infection [3, 56–58]. Also, overexpression of strigolactone biosynthesis genes such as *GmMAX1a, GmMAX3b, GmMAX4a, and GmMAX2a* are extremely correlated with the augmented nodule number and nodule development, whereas knocking down these genes diminishes nodulation [28, 54, 59, 60]. As well as, Ahmad et al. (2020) [37] reported that the overexpression of *GmMaT2* in the *G. max* hairy roots system enhances the expression of early nodulation genes such as *DMI2α, DMI3α, NSP2β, NSP1α, NFR5α*, and *NFR1α* but compromised in *GmMaT2* knockdown compared with the control. So, we propose that both hormones (such as strigolactones or brassinosteroids) are likely to act linked with the autoregulation of the nodulation (AON) system, having a role in the promotion of nodule formation and maintenance of meristematic activity during nodule development [25, 26, 28, 54, 59, 61]. In general, the overexpression of the *GsMaT2* gene from wild soybean led to increased transcription of nodulation signaling and SL biosynthesis genes. This finding points to isoflavone-7-O-beta-glucoside 6″-O-malonyltransferase playing critical roles in root development and nodulation in soybean. Our results demonstrate that the overexpression of the *GsMaT2* gene studied here significantly increased nodulation and root growth compared to control hairy roots of soybean overexpressing GUS. Finally, the main findings are summarised in Fig. 7.

## Conclusions

In summary, this study focuses on cloning *GsMaT2* from wild soybean (*G. soja*), encoding isoflavone-7-O-beta-glucoside 6″-O-malonyltransferase, over-expressed it in hairy root systems of the cultivated soybean (*G. max*), and assessing the root architecture, growth, and nodulation. Substantial differences in root growth, root nodulation, expression levels of nodulation signaling, and SL biosynthesis genes were observed. We further used bioinformatics and the putative expression analysis as tools to predict the role of *GsMaT2* in root and nodule development. Our data suggest that the *GsMaT2* gene promotes root development and nodulation signaling by activating nodulation signaling and SL synthetic genes. These findings prove that the overexpression of the *GsMaT2* gene could affect the nodulation signaling pathway and nodulation. Our study clarifies our understanding of the exact function of the *GsMaT2* gene in root and nodulation, including the role of increased *GsMaT2* expression in both root and nodules, in conjunction with the nodulation signaling and SL synthetic genes that are crucial for legume nodulation production.

## Material and methods

### Phylogenetic analysis

The full-length cDNA of *GsIMaT2* was retrieved from GenBank ID: *XM_028358532.1* and Phytozome ID: *GlysoPI483463.18G200800*.. Additionally, the *GsIMaT2* product was identified as (A0A445FY23) from the UniProt database [62], which was then used as a query in a blastp search with a cutoff e-value <1e− 04 to retrieve authentic homologous proteins from *Glycine soja*, *Glycine max*, *Nicotiana tabacum*, *Arabidopsis thaliana*, *Salvia splendens* and *Amborella trichopoda* [63]. All retrieved protein sequences were aligned by Decipher [64]. The ProtTest v3.4.2 was used to select the best-fitted amino acid substitution model based on the lowest Bayesian information criterion (BIC) score [65]. Afterwards, the Bayesian phylogenetic tree was constructed via MrBayes v3.2.6. with the Jones–Taylor–Thornton (JTT) amino acid substitution model with invariant sites, discrete gamma model, and (+ F) for the empirical equilibrium of amino acid frequencies [66]. Finally, the phylogenetic tree was introduced to the iTOL (Interactive Tree of Life) web tool for visualization [67].

### *In-silico* differential gene expression analysis

Tissue-specific expression data from twenty-eight soybean tissues after inoculation and fertilization (e.g., Root Hair 12HAI, Roothair_12HAImock, Root Hair 24 HAI, Roothair_24HAImock, Root Hair 48 HAI, Roothair_48HAImock, Root Hair 48 HAI Stripped, SAM, Flower, Green_Pods, Leaves, Nodule, Root, Root_tip, Young Leaf, Flower, One CM Pod, Pod Shell (10-13 DAF), Pod Shell (14 - 17 DAF), Nodule, Root, Seed 10 - 13 DAF, Seed 14 - 17 DAF, Seed 21 DAF, Seed 25 DAF, Seed 28 DAF, Seed 35 DAF and Seed 42 DAF) were extracted from public RNA-Seq Atlas of soybean (http://bar.utoronto.ca/eplant_soybean/). Additionally, *GsIMaT2* predicted subcellular localization was inferred from its Arabiposis homologous genes as retrieved from the Arabidopsis Information Resource (https://phytozome.jgi.doe.gov/pz/portal.html#!info?alias=Org_Athaliana).. Ultimately, the image that showed the subcellular localization was built using Cell Electronic Fluorescent Pictograph Browsers (Cell eFP: http://bar.utoronto.ca/cell_efp/cgi-bin/cell_efp.cgi) [68, 69].

### RNA extraction and cDNA synthesis

Total RNA from three biological replicates of *G. soja* was extracted from roots using TRIzol reagent (Invitrogen, CA, United States) according to the manufacturer’s methods and instructions. Also, total RNA was extracted from twelve biological replicates of *G. max* hairy roots and nodulated roots upon 10 and 20 days of rhizobial inoculation. Total RNA samples were treated with DNase I (Takara, China). RNA quality was examined on 1.2% Agarose gels, and the purity and concentration were analyzed using a Nano-Photometer spectrophotometer (IMPLEN, CA, USA). cDNA synthesis for gene cloning and qRT-PCR was performed with a 10 μg total RNA pool produced by mixing equal volumes of the three RNA replicates in a tube using a commercial reverse transcription kit (M-MLV, China) according to the manufacturer’s protocol [24, 68, 70, 71].

### Cloning of full-length isoflavone-7-O-beta-glucoside 6″-O-malonyltransferase (*GsIMaT2*) gene

The *GsIMaT2* full-length cDNAs (GenBank ID: ON520655.1) with a length of 1536 bp was obtained by PCR amplification using short and long gene-specific primers designed based on the transcriptome sequencing of *G. soja* from soybean database (https://phytozome.jgi.doe.gov/pz/portal.html). Root cDNA was used as a template for the first PCR, which was performed with short primers, such as *GsIMaT2* forward 5′-ATGGCAGTGGAAAATATCAAAGTC − 3′and reverse 5′- TCACTCATCTTTCAGTCCTCCATG − 3′ with the KOD-Plus DNA polymerase (Toyobo, Japan) with the following cycling conditions: an initial step of 4 min at 95 °C followed by 34 cycles of denaturation for 10s at 98 °C; 30s at 60 °C and an extension for 2 min at 68 °C, and a final extension step for 11 min at 68 °C. The first PCR products were used as templates for PCR cloning using long primers, such as *GsIMaT2* forward 5′-GGGGACAAGTTTGTACAAAAAAGCAGGCTTCATGGCAGTGGAAAATATC-3′and reverse 5′-GGGGAC CACTTTGTACAAGAAAGCTGGGTTCACTCATCTTTCAGTCC-3′ with the and KOD-Plus DNA polymerase. The amplified PCR products were purified using (QIAEX II Gel Extraction Kit, China) and cloned into the Gateway entry vector pDONR221 using BP Clonase (Gateway™ BP Clonase™ II Enzyme mix, Invitrogen) [24, 68, 70, 71]. The resulting pDONR221 construct harbouring target gene was sequenced, and the LR Clonase (Gateway™ LR Clonase™ Enzyme mix, Invitrogen) was used for recombination into the destination vector pB2GW7 for *G. max* hairy root transformation to produce composite soybean plants. Sanger sequencing confirmed that all final constructs contained *GsIMaT2* cDNAs. The construct was introduced into *Agrobacterium rhizogenes* strain “K599” by direct electroporation.

### Soybean hairy root transformation and rhizobial inoculation

Seeds of soybean cv. “Tianlong 1” were surface sterilized by placing 150 seeds in 15 × 100 mm Petri dishes in a single layer. The plates were placed inside a 1000-mL beaker with 200 mL of commercial bleach. Ten microliters of concentrated (12 N) HCl were applied dropwise to the beaker’s internal wall; the container was sealed with a plastic cover and kept overnight (16 hours). The following morning, the sterilized seeds were germinated in sterile vermiculite in a growth chamber (12-h photoperiod, 28 °C day/25 °C night, and 70% humidity) for a few days until hairy root transformation.

Recombinant *A. rhizogene*s were grown for two days at 28 °C on solid LB media supplemented with 50 μg/mL of each streptomycin and spectinomycin. An individual colony of each construct was inoculated into 1 mL of liquid LB medium with the same antibiotics and grown at 28 °C under 200 rpm agitation overnight. After 24 h, the liquid cultures were transferred into a 250-mL conical flask containing 50 mL of LB media supplemented with the same antibiotics and grown in a shaker at 28 °C until an optical density (OD_600_) of 0.6–8.0 was reached. Overnight cell cultures were harvested by centrifugation at 5000 rpm for 10 min at 4 °C, and the pellet was re-suspended to an OD of half-strength B5 medium containing 3% sucrose. Healthy and vigorous seedlings with unfolded green cotyledons were inoculated with *A. rhizogenes* strain K599 harboring the binary vectors by injecting the hypocotyls proximal to the cotyledon with the bacterial suspension. The infected seedlings were then transplanted into 10 cm x10cm × 8.5 cm pots with vermiculite with the infection site buried, and each pot was covered with a transparent plastic bag to retain humidity.

The rhizobial inoculations of hairy roots were carried out with 15-day-old plants (10 days after root transformation). A culture of *B. japonicum* strain “USDA-110” was cultured onto yeast extract mannitol agar (YMA) at 28 °C. After ten days of hairy root emergence, the optical density (OD_600_) of a rhizobium liquid YM culture was adjusted to 0.08-1.0, and about 50 mL were applied to each pot. After ten and twenty days of rhizobial inoculation, the plants (*n* = 10–12) with well-developed hairy roots and nodules were photographed and harvested for measurements and RNA isolation to assess gene expression. Hairy root systems from each plant were considered independent transformation events [24, 28].

### Quantitative real-time PCR analyses

Quantitative real-time PCR (qRT-PCR) was performed using an iQ™5 Multicolor Real-Time PCR Detection System (Bio-Rad) with SYBR Green fluorescence (and ROX as a passive reference dye; Newbio Industry, China) in a total reaction volume of 20 μL, as described previously [24, 68, 70, 71]. Gene-specific primers for *GmActin* as a reference gene and *GsIMaT2* were used. Primers were designed with the IDTdna tool (https://eu.idtdna.com/scitools/Applications/RealTimePCR/), and their sequences are listed in Additional file 5: Table S1. Additionally, gene-specific primers for nineteen *G. max* genes involved in early nodulation signaling genes such as *GmDMI2a, GmDMI2B, GmDMI3a, GmDMI3b, GmNSP1a, GmNSP1B, GmNSP2a, GmNSP2B, GmNINa, GmNINB, GmNRF1, GmNRF5* and *GmEnod40*, and SL synthetic genes such as *GmMAX1a, GmMAX1B, GmMAX2, GmMAX3, GmMAX4a,* and *GmMAX4B*.are listed in Additional file 5: Table S1. The amplicon sizes were designed between 145 and 160 bp. The quantitative RT-PCR standard conditions were: 95 °C for 3 min, 34 amplification cycles (95 °C for 10s, 58 °C or 60 °C for 30s, and 72 °C for 20s), followed by 65 °C for 5 s and 95 °C for 5 s). The relative expression levels were calculated by comparing the target genes’ cycle thresholds (CTs) with the reference gene *GmActin*. Data quantification was carried out with the Bio-Rad IQ™ 5 Multicolor Real-Time Manager software using the 2^-ΔΔCt^ method [71–73] and *GmActin* as a reference housekeeping gene for normalization. Values are presented as means ± SE of three different RNA pool replicates*.*

### Extraction and HPLC analysis of isoflavones from transgenic in vitro hairy root

The right method to reduce technical variability throughout a sampling collection and preparation is essential to stop cell metabolism and avoid leaking metabolites during the various preparation steps before the actual metabolite extraction [24, 68, 71–73]. Therefore, three replicates of fresh hairy roots from each *GsIMaT2* overexpression and Gus as control were frozen immediately on liquid nitrogen (L.N). In the laboratory, the transgenic hairy roots from each sample were homogenized into a powder in L. N with a mortar and pestle, after which the plant material (ca. 250 mg) was directly soaked in 2 ml of 80% methyl alcohol, then sonicated for 30 min, and kept on a rotator shaker at 4 °C for overnight extraction. The Next day, after centrifugation for 30 min at 12000 rpm at 4 °C, the surface layer was filtered with 0.22 μM Millipore filters for analysis. The supernatant was pipette into a fresh crimp vial amber glass, 1.5 ml screw-top vials with silicone/PTFE septum lids (http://www.sigmaaldrich.com). Moreover, the crimp vial was placed on the auto-sampler of the HPLC system for HPLC analysis. The isoflavonoids from G*sMaT2* and Gus as control were analyzed with HPLC (Shimadzu SPD M-20A) with a DAD detector (Shimadzu, Kyoto, Japan) using Inertsil ODS-3 column (250 mm × 4.6 mm × 5 μm) at a flow rate of 1.0 ml min–1 and a 10 μl injection volume, as described previously [6, 16, 74]. The concentration of isoflavone was calculated using the standard curve [41].

### Statistical analyses

The Student’s *t*-test analyzed Soybean hairy root measurements to estimate the effects of gene overexpression and time on the number of nodules, nodule fresh weight (gram), fresh root weight (gram), and root length (cm) compared to the control roots (*GUS*-overexpressing hairy roots). Each column represents the mean ± SD of the parameter, and statistical significance was based on the Student’s *t*-test (*P < 0.05; ** *P* < 0.01) with GUS-overexpressing hairy roots as control.

## Supplementary Information

**Additional file 1: Fig. S1.** Bayesian phylogenetic analysis for the wild soybean phenolic glucoside malonytransferase *GsIMaT2* (A0A445FY23) and its paralogous from *Glycine soja* and its orthologous from *Glycine soja*, *Glycine max*, *Nicotiana tabacum*, *Arabidopsis thaliana*, and *Salvia splendens*. The uniport IDs for various homologous proteins are labelled on the tree after the plant abbreviation.

**Additional file 2: Fig. S2.** Multiple sequence alignment. The deduced amino acid sequence of *GsIMaT2* was aligned with homologue *GmIMaT2* identified from the BLASTP analysis. *GsIMaT2* (**UTK46214.1**): isoflavone-7-O-beta-glucoside 6″-O-malonyltransferase proteins from *G. soja*; *GmIMaT2* (**XP_003552518.1**): isoflavone-7-O-beta-glucoside 6″-O-malonyltransferase proteins from *G. max*. *GsIMaT2* has two amino acid differences from *GmIMaT2* at Q75L and D192Y. Q, L, D and Y represent the abbreviation of amino acid, while 75 and 192 represent the position of amino acid in the protein sequence. For multiple sequence alignment analyses we used CLUSTALW (https://www.genome.jp/tools-bin/clustalw).

**Additional file 3: Fig. S3.** Comparing the sequence and secondary structure of the deduced amino acid sequence of isoflavone-7-O-beta-glucoside 6″-O-malonyltransferase *GsIMaT2* (**I1N4C1**) with its authentic homologous from the cultivated soybean *GmIMaT2* (**A0A445FY23**) as retrieved from a BLASTP search. The *GsIMaT2* has two amino acid different from *GmIMaT2* at Q75L and D192Y. The proteins secondary structures were deduced via alphafold (Jumper et al., 2021) and plotted on the alignment through ESPript 3.0 (Robert and Gouet, 2014).

**Additional file 4: Fig. S4.** Putative tissue-specific expression and subcellular localisations of *GsIMaT2* gene based on soybean tissues and Arabidopsis protein localization at different cell organs. (A and B) Soybean tissue-specific expression was built using public RNA-Seq Atlas of soybean from twenty eight soybean tissues after inoculation and fertilization using (http://bar.utoronto.ca/eplant_soybean/). (C) Cell sub-cellular localizations profile images were built using Cell Electronic Fluorescent Pictograph Browsers (Cell eFP browsers. The blue arrow points the expression scale (the more intense red color, the more gene expression), http://bar.utoronto.ca/cell_efp/cgi-bin/cell_efp.cgi.

**Additional file 5: Table S1.** List of *Glycine max* genes involved in strigolactone biosynthesis and nodulation signaling pathway way and primer pairs used for qRT-PCR.

## Data Availability

All data generated or analyzed during this study are included in this published article and its supplementary information files. The datasets used and/or analyzed during the current study are available from the corresponding author on reasonable request. GenBank accession number: Glycine soja isoflavone-7-O-beta-glucoside 6″-O-malonyltransferase (GsIMaT2, GenBank: ON520655.1) https://www.ncbi.nlm.nih.gov/nuccore/ON520655.1.
