# Supplementary figures and images for: Constitutive overexpression of GsIMaT2 gene from wild soybean enhances rhizobia interaction and increase nodulation in soybean (Glycine max)
Source: BMC Plant Biol. 2022 Sep 9;22:431. doi: 10.1186/s12870-022-03811-6 (PMC9461152; doi:10.1186/s12870-022-03811-6)

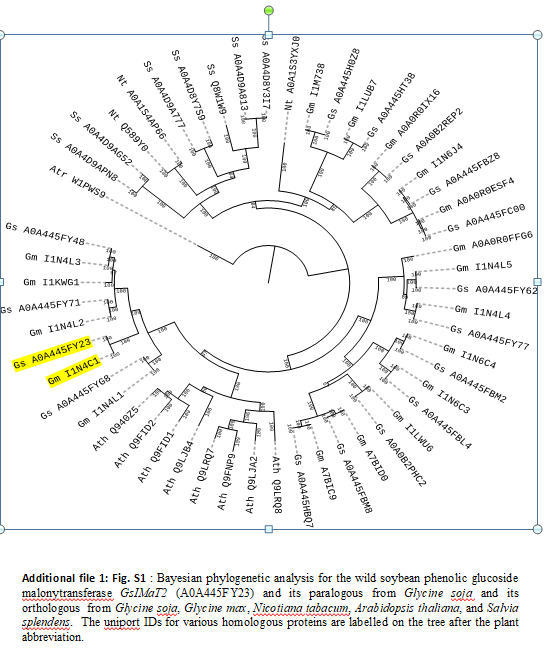

Supplement: Supplementary file 1 — Additional file 1: Fig. S1. Bayesian phylogenetic analysis for the wild soybean phenolic glucoside malonytransferase GsIMaT2 (A0A445FY23) and its paralogous from Glycine soja and its orthologous from Glycine soja, Glycine max, Nicotiana tabacum, Arabidopsis thaliana, and Salvia splendens. The uniport IDs for various homologous proteins are labelled on the tree after the plant abbreviation. [file 12870_2022_3811_MOESM1_ESM.docx]

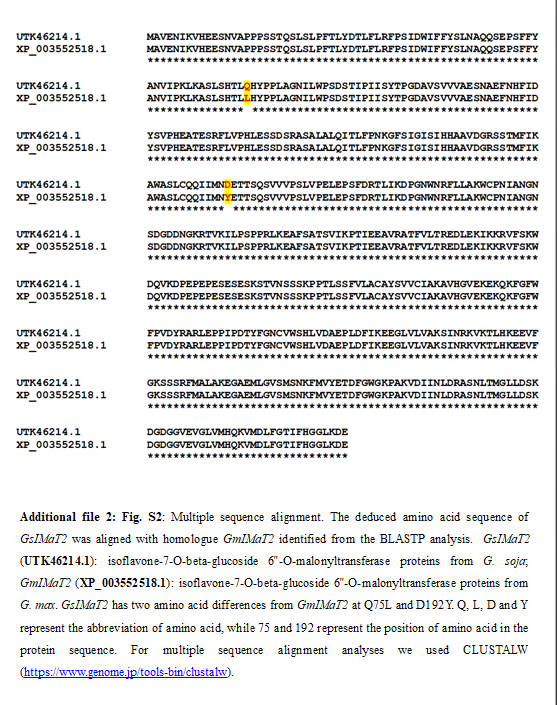

Supplement: Supplementary file 2 — Additional file 2: Fig. S2. Multiple sequence alignment. The deduced amino acid sequence of GsIMaT2 was aligned with homologue GmIMaT2 identified from the BLASTP analysis. GsIMaT2 (UTK46214.1): isoflavone-7-O-beta-glucoside 6″-O-malonyltransferase proteins from G. soja; GmIMaT2 (XP_003552518.1): isoflavone-7-O-beta-glucoside 6″-O-malonyltransferase proteins from G. max. GsIMaT2 has two amino acid differences from GmIMaT2 at Q75L and D192Y. Q, L, D and Y represent the abbreviation of amino acid, while 75 and 192 represent the position of amino acid in the protein sequence. For multiple sequence alignment analyses we used CLUSTALW (https://www.genome.jp/tools-bin/clustalw). [file 12870_2022_3811_MOESM2_ESM.docx]

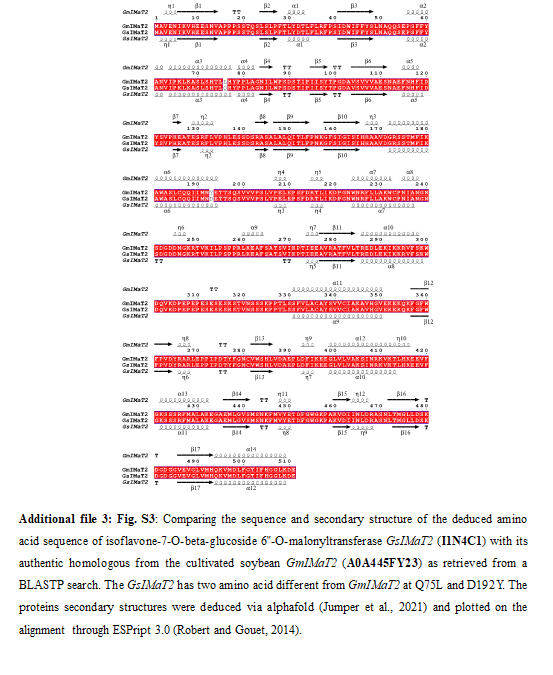

Supplement: Supplementary file 3 — Additional file 3: Fig. S3. Comparing the sequence and secondary structure of the deduced amino acid sequence of isoflavone-7-O-beta-glucoside 6″-O-malonyltransferase GsIMaT2 (I1N4C1) with its authentic homologous from the cultivated soybean GmIMaT2 (A0A445FY23) as retrieved from a BLASTP search. The GsIMaT2 has two amino acid different from GmIMaT2 at Q75L and D192Y. The proteins secondary structures were deduced via alphafold (Jumper et al., 2021) and plotted on the alignment through ESPript 3.0 (Robert and Gouet, 2014). [file 12870_2022_3811_MOESM3_ESM.docx]

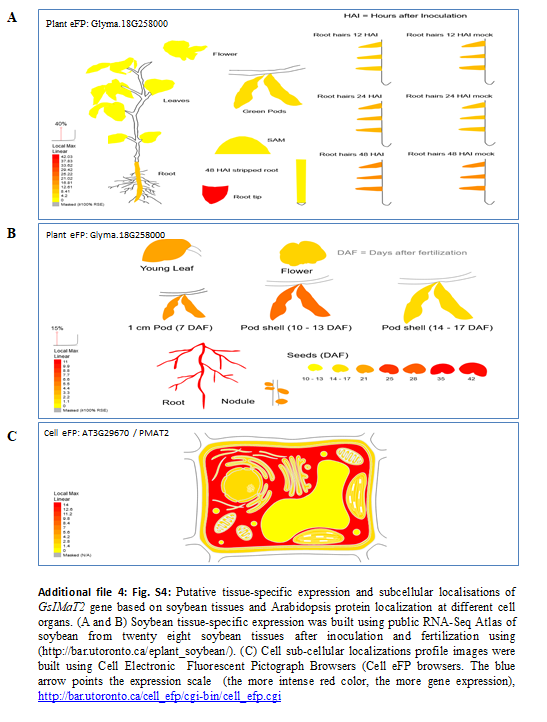

Supplement: Supplementary file 4 — Additional file 4: Fig. S4. Putative tissue-specific expression and subcellular localisations of GsIMaT2 gene based on soybean tissues and Arabidopsis protein localization at different cell organs. (A and B) Soybean tissue-specific expression was built using public RNA-Seq Atlas of soybean from twenty eight soybean tissues after inoculation and fertilization using (http://bar.utoronto.ca/eplant_soybean/). (C) Cell sub-cellular localizations profile images were built using Cell Electronic Fluorescent Pictograph Browsers (Cell eFP browsers. The blue arrow points the expression scale (the more intense red color, the more gene expression), http://bar.utoronto.ca/cell_efp/cgi-bin/cell_efp.cgi. [file 12870_2022_3811_MOESM4_ESM.docx]
