# Supplementary material for: Constitutive overexpression of GsIMaT2 gene from wild soybean enhances rhizobia interaction and increase nodulation in soybean (Glycine max)
Source: BMC Plant Biol. 2022 Sep 9;22:431. doi: 10.1186/s12870-022-03811-6 (PMC9461152; doi:10.1186/s12870-022-03811-6)
Supplement: Supplementary file 5 — Additional file 5: Table S1. List of Glycine max genes involved in strigolactone biosynthesis and nodulation signaling pathway way and primer pairs used for qRT-PCR. [file 12870_2022_3811_MOESM5_ESM.doc]

[**Additional file1: Table S**](http://dnaresearch.oxfordjournals.org/lookup/suppl/doi:10.1093/dnares/dst051/-/DC1)**1.** List of *Glycine max* genes involved in strigolactone biosynthesis and nodulation signaling pathway way and primer pairs used for qRT-PCR.

| **Gene name** | **Primer name** | **Primer sequence** | **PCR product (bp)** |
| --- | --- | --- | --- |
| *GmMAX1a* | *GmMAX1a-F* | 5ʹ- CCTCTCTAGTCCCCATTCAGTTT-3ʹ | 150 |
|  | *GmMAX1a-R* | 5ʹ- TGTGTTGGTTGATGAAATCTGATAC-3ʹ |  |
| *GmMAX1b* | *GmMAX1b-F* | 5ʹ- TACCGCAAATATTCTTCAGACC-3ʹ | 155 |
|  | *GmMAX1b-R* | 5ʹ- ACCAGATTCTATCCCTTTCACC-3ʹ |  |
| *GmMAX2* | *GmMAX2-F* | 5ʹ- TGTTTGCTCGCCAGGACTC-3ʹ | 160 |
|  | *GmMAX2-R* | 5ʹ- CACCATCACCCGGTTCATC-3ʹ |  |
| *GmMAX3* | *GmMAX3-F* | 5ʹ- CAGGCTACTCACCGTGTCTT-3ʹ | 152 |
|  | *GmMAX3-R* | 5ʹ- GGTATCCGTTACAGCCCAAT-3ʹ |  |
| *GmMAX4a* | *GmMAX4a-F* | 5ʹ- CTTTCACCACCTTGCCTTCA-3ʹ | 148 |
|  | *GmMAX4a-R* | 5ʹ- TGGTTTCTTTCCGTTCCTCC-3ʹ |  |
| *GmMAX4b* | *GmMAX4b-F* | 5ʹ- GAAATAAAGGCATCTACAAAGGGAA-3ʹ | 150 |
|  | *GmMAX4b-R* | 5ʹ- ATGGTGGTGGTGGTGGCAGT-3ʹ |  |
| *GmNINa* | *GmNINa-F* | 5ʹ- TAACATGCGATGCTGATCTTG-3ʹ | 150 |
|  | *GmNINa-R* | 5ʹ- TGATTTAGAGGCGAAGCTTGA-3ʹ |  |
| *GmNINb* | *GmNINb-F-* | 5ʹ- CATGGAGTCGACGCAAATAA-3ʹ | 160 |
|  | *GmNINb-R* | 5ʹ- TCAAGTACCCAACAGCAATC-3ʹ |  |
| *GmNFR5* | *GmNFR5-F* | 5ʹ- TTCCCTTTCTTCCTCTCCAC-3ʹ | 149 |
|  | *GmNFR5-R* | 5ʹ- GCATGAAAAGTTTGTTCTATTGTC-3ʹ |  |
| *GmNSP1a* | *GmNSP1a-F* | 5ʹ- CAACACTTATCTTCTTCTCCAACT-3ʹ | 160 |
|  | *GmNSP1a-R* | 5ʹ- GGAAGCATTTGCTATGTTGTTAGG-3ʹ |  |
| *GmNSP1b* | *GmNSP1b-F* | 5ʹ- ATCCTCGTCTTCTTCCAAATAC-3ʹ | 155 |
|  | *GmNSP1b-R* | 5ʹ- GGGAGAAGGAGTAGGAGTAAT-3ʹ |  |
| *GmNSP2a* | *GmNSP2a-F* | 5ʹ- GAACTTACCGCACCTTAGTT-3ʹ | 155 |
|  | *GmNSP2a-R* | 5ʹ- GATGCAGCGACTCCATAAA-3ʹ |  |
| *GmNSP2b* | *GmNSP2b-F* | 5ʹ- AATCATTGCCAAGCGAAGCT-3ʹ | 149 |
|  | *GmNSP2b-R* | 5ʹ- AGTCCAAAGCGAGGCAGAGA-3ʹ |  |
| *GmDMI2a* | *GmDMI2a-F* | 5ʹ- GTCCTCAGTGGCCTTGACATT-3ʹ | 160 |
|  | *GmDMI2a-R* | 5ʹ- ACACCCTTTTGCCTGCTTTG-3ʹ |  |
| *GmDMI2b* | *GmDMI2b-F* | 5ʹ- ATTCACGAGCACACTGTGCCT-3ʹ | 158 |
|  | *GmDMI2b-R* | 5ʹ- CCAAAATCTGCAACCTTTCC-3ʹ |  |
| *GmDMI3α* | *GmDMI3α-F* | 5ʹ- AGTGTTTGGAGCACCGCAATC-3ʹ | 147 |
|  | *GmDMI3α-R* | 5ʹ- TCAAACAAGTCAAATATACGTGGTG-3ʹ |  |
| *GmDMI3b* | *GmDMI3b-F* | 5ʹ- TTGTCCATAGGGAGGGTAAT-3ʹ | 159 |
|  | *GmDMI3b-R* | 5ʹ- ATGAGTAGAAGGGTAGCTAGAG-3ʹ |  |
| *GmNFR1* | *GmNFR1-F* | 5ʹ- ATTCACGAGCACACTGTGCCT-3ʹ | 158 |
|  | *GmNFR1-R* | 5ʹ- CCAAAATCTGCAACCTTTCC-3ʹ |  |
| *GmEnod40* | *GmEnod40-F* | 5ʹ- GAAAGGGGTGTGAGAGGAGAG-3ʹ | 160 |
|  | *GmEnod40-R* | 5ʹ- CGCCACTCAAGAAAGAATGTT-3ʹ |  |
| *GmACTIN* | *GmACTIN-F* | 5ʹ- CTTCCCTCAGCACCTTCCAA-3ʹ | 158 |
|  | *GmACTIN-R* | 5ʹ- GGTCCAGCTTTCACACTCCAT-3ʹ |  |
| *GsIMaT2* | *GsIMaT2-F* | 5ʹ-CTTCAAAGCCACCAACTTTATC-3ʹ | 145 |
|  | *GsIMaT2-R* | 5ʹ- GGCTCTAACCTAGCCCTATAA-3ʹ |  |
